# Association between iridocyclitis and immune-related disease: A 2-sample Mendelian randomization study

**DOI:** 10.1097/MD.0000000000040663

**Published:** 2024-11-29

**Authors:** Yao Yao, Qian Wang, Wenbin Wei

**Affiliations:** a Beijing Tongren Eye Center, Beijing Tongren Hospital, Beijing Ophthalmology and Visual Science Key Lab, Beijing Key Laboratory of Intraocular Tumor Diagnosis and Treatment, Capital Medical University, Beijing, China.

**Keywords:** anterior uveitis, immune-related disease, iridocyclitis, Mendelian randomization, risk factors

## Abstract

The genetic basis of iridocyclitis, an inflammatory eye disease, remains poorly understood, particularly in relation to autoimmune diseases. This study aimed to explore the causal associations between 6 immune-related diseases and iridocyclitis using Mendelian randomization (MR). A total of 230 single nucleotide polymorphisms (SNPs) significantly associated with systemic lupus erythematosus, ankylosing spondylitis (AS), rheumatoid arthritis (RA), Graves disease (GD), Crohn disease (CD), and allergic contact dermatitis were identified based on stringent MR assumptions. These SNPs served as instrumental variables to estimate the causal effect of each autoimmune disease on iridocyclitis risk. The analysis utilized the inverse variance weighted method, complemented by sensitivity analyses including MR-Egger regression and leave-one-out testing to assess pleiotropy and robustness. The MR analysis revealed significant associations between genetically predicted AS (odds ratio [OR]: 1.544, 95% confidence interval [CI]: 1.494–1.595, *P* = 1.99 × 10^−226^), RA (OR: 1.207, 95% CI: 1.052–1.385, *P* = .003), and CD (OR: 1.654, 95% CI: 1.263–2.166, *P* = 2.54 × 10⁻⁶) with an increased risk of iridocyclitis. Conversely, higher genetically predicted GD was associated with a decreased risk of iridocyclitis (OR: 0.763, 95% CI: 0.674–0.865, *P* = .0002). Although systemic lupus erythematosus and allergic contact dermatitis appeared to have a protective effect, these results were not statistically significant, and no causal relationship could be established. Heterogeneity was observed among the SNPs, but no significant horizontal pleiotropy was detected. This study identifies potential genetic links between AS, RA, CD, GD, and the risk of iridocyclitis, providing new insights into the genetic underpinnings of this eye disease. The results support the need for further investigation into the genetic and molecular mechanisms underlying these associations.

## 1. Introduction

Iridocyclitis, also referred to as anterior uveitis, is an inflammatory condition targeting the anterior segment of the uveal tract, specifically the iris and ciliary body. It stands as the most prevalent form of iridocyclitis and represents a significant cause of ocular morbidity worldwide, with global incidence rates of anterior uveitis ranging from 12 to 52 cases per 100,000 individuals annually.^[1–3]^ Iridocyclitis may occur as an isolated ocular event or in association with systemic diseases, particularly those involving immune dysregulation. Central to its pathogenesis are pro-inflammatory cytokines, including interleukins (IL-1, IL-6, IL-17), tumor necrosis factor-alpha (TNF-α), and interferon-gamma, which orchestrate immune cell activation and tissue infiltration.^[4,5]^ These cytokines initiate a cascade of events involving chemokines such as CCL2 and CXCL8, facilitating the recruitment of leukocytes and perpetuating local inflammation within the uveal tissue. Immune cells play pivotal roles in iridocyclitis, with effector T lymphocytes, particularly Th1 and Th17 subsets, significantly contributing to disease severity.^[6,7]^ Th1 cells produce IFN-γ, driving macrophage activation and tissue damage, while Th17 cells release IL-17, perpetuating chronic inflammation and tissue remodeling.^[8,9]^ Activated macrophages further exacerbate inflammation through the secretion of pro-inflammatory cytokines and reactive oxygen species, contributing to oxidative stress and subsequent damage to retinal and choroidal tissues.

The relationship between iridocyclitis and immune-related diseases is marked by shared genetic predispositions^[10]^ and overlapping immunopathogenic mechanisms. Iridocyclitis frequently coexists with autoimmune disorders such as rheumatoid arthritis, ankylosing spondylitis, and inflammatory bowel disease, reflecting common genetic factors like human leukocyte antigens (HLA) alleles and dysregulated immune responses. These conditions often present with anterior iridocyclitis as a predominant manifestation, though posterior and paniridocyclitis forms are also observed in certain systemic diseases like Behçet disease.^[11,12]^ Despite this, past studies have yielded discordant results regarding the relationship between these immune-related diseases, with the magnitude of reported associations varying considerably. Thus, a causal relationship between immune-related diseases and iridocyclitis remains unestablished.

Mendelian randomization (MR) is an epidemiological method that utilizes genetic variants as instrumental variables (IVs) to infer causal relationships between modifiable risk factors and health outcomes.^[13,14]^ This approach relies on the principle of allelic randomization at conception, which ensures that genetic variants are not confounded by external factors that typically affect observational studies. The methodology hinges on specific assumptions: the genetic variants must be strongly associated with the risk factor (relevance), independent of confounders (independence), and must affect the outcome solely through the risk factor (exclusion restriction).

## 2. Methods

### 2.1. Mendelian randomization

**Figure 1.**
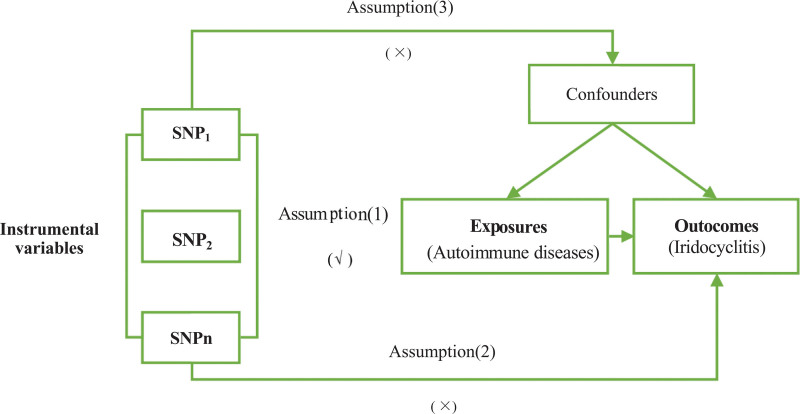
Overview of Mendelian randomization. SNP = single nucleotide polymorphisms.

The MR analysis was conducted based on 3 core assumptions: the genetic variants were robustly associated with the exposure; the genetic variants influenced the outcome exclusively through the exposure; and the association was independent of any potential confounders (Fig. 1). Generally, the use of Genome-Wide Association Studies (GWAS) aggregate data effectively satisfies the first assumption, while the latter 2 assumptions may not always hold. Since genetic variation is not affected by potential confounders or outcomes, any observed effect on the outcome must occur through its impact on the exposure, which is itself influenced by the genetic variation. Consequently, associations identified through MR are considered causal.

### 2.2. Data source description

This MR study utilized summary-level data from a published GWAS dataset on iridocyclitis and immune-related diseases, with appropriate patient consent and ethical approval obtained. Eight immune-related diseases were selected as exposure variables, and iridocyclitis was chosen as the outcome variable. The summary data for ankylosing spondylitis (AS, finn-b-M13_ANKYLOSPON), autoimmune hyperthyroidism (Grave disease, GD, finn-b-AUTOIMMUNE_HYPERTHYROIDISM), rheumatoid arthritis (RA, ebi-a-GCST90013534), systemic lupus erythematosus (SLE, ebi-a-GCST003156), Crohn disease (CD, finn-b-K11_CROHN), allergic contact dermatitis (ACD, finn-b-L12_ALLERGICCONTACT), and iridocyclitis (finn-b-H7_IRIDOCYCLITIS) were sourced from the official GWAS repository (https://gwas.mrcieu.ac.uk). All GWAS datasets represent European populations (Table 1).

**Table 1:** Phenotype source and description.

Phenotype	PMID	First author	Sample size	Cases (n)	Controls (n)	Population
Systemic lupus erythematosus	26502338	Bentham J (2015)	14,267	5201	9066	European
Ankylosing spondylitis	NA	NA (2021)	166,144	1462	164,682	European
Rheumatoid arthritis	33310728	Ha (2020)	58,284	14,361	43,923	European
Autoimmune hyperthyroidism	NA	NA (2021)	173,938	962	172,976	European
Crohn disease	NA	NA (2021)	212,356	2056	210,300	European
allergic contact dermatitis	NA	NA (2021)	200,944	2204	198,740	European

NA = not applicable.

### 2.3. Statistical analysis

All statistical analyses were performed using R software (version 4.0.2). To investigate the causal relationship between immune-related diseases and iridocyclitis, we selected SNPs associated with immune-related diseases as instrumental variables and filtered for SNPs significantly associated with disease across the genome (*P* < 5 × 10 ^−8^). To minimize confounding, linkage disequilibrium analysis was conducted to ensure that selected SNPs were independent of other loci. SNPs exhibiting linkage imbalance (*r*^2^ < 0.01) were excluded, and independent SNPs were identified across 5 immune-related diseases to assess causality.

In this study, inverse variance weighting (IVW) with random effects served as the primary analysis method, offering the most precise estimates but with sensitivity to pleiotropy. To address this, the weighted median method and MR-Egger regression were employed for sensitivity analysis. The weighted median method provides consistent causal estimates, assuming that at least 50% of the weight comes from valid SNPs. MR-Egger regression, meanwhile, detects potential pleiotropic effects and adjusts estimates accordingly.^[15]^ Cochrane *Q* value was calculated to assess heterogeneity. A *P* value >.05 indicated no significant heterogeneity among the SNPs; otherwise, a random effects model was used for recalculations. The leave-one-out method was applied for sensitivity analysis, where the causal effect of the remaining SNPs on the outcome was analyzed sequentially after removing each SNP to evaluate the impact of individual SNPs on the results.

## 3. Results

### 3.1. Selection of instrumental variables

A total of 230 SNPs significantly associated with immune-related diseases were identified based on the 3 core assumptions of MR and the criteria for selecting IVs. The SNPs used as IVs were distributed as follows: 48 SNPs for SLE, 43 SNPs for AS, 106 SNPs for RA, 10 SNPs for GD, 12 SNPs for CD, and 11 SNPs for ACD. All selected SNPs were significantly associated with their corresponding autoimmune diseases (AID) at a threshold of *P* < 5 × 10⁻⁸, as detailed in Table 2.

**Table 2:** MR analysis of immune-related disease and allergic rhinitis.

Phenotype	Method	No. SNP	*β*	Standard error	OR	95% CI	*P* value
Systemic lupus erythematosus	IVW	48	−0.044	0.045	0.957	0.877–1.044	.3292
Ankylosing spondylitis	IVW	43	0.434	0.014	1.544	1.494–1.595	1.99 × 10^−226^
Rheumatoid arthritis	IVW	106	0.188	0.064	1.207	1.052–1.385	.0033
Autoimmune hyperthyroidism	IVW	10	−0.270	0.073	0.763	0.674–0.865	.0002
Crohn disease	IVW	12	0.503	0.107	1.654	1.263–2.166	2.54 × 10^−6^
Allergic contact dermatitis	IVW	11	−0.149	0.173	0.862	0.629–1.181	.3910

CI = confidence interval, IVW = inverse variance weighted, MR = Mendelian randomization, OR = odds ratio, SNP = single nucleotide polymorphisms.

### 3.2. Causal effects of immune-related diseases on iridocyclitis

**Figure 2.**
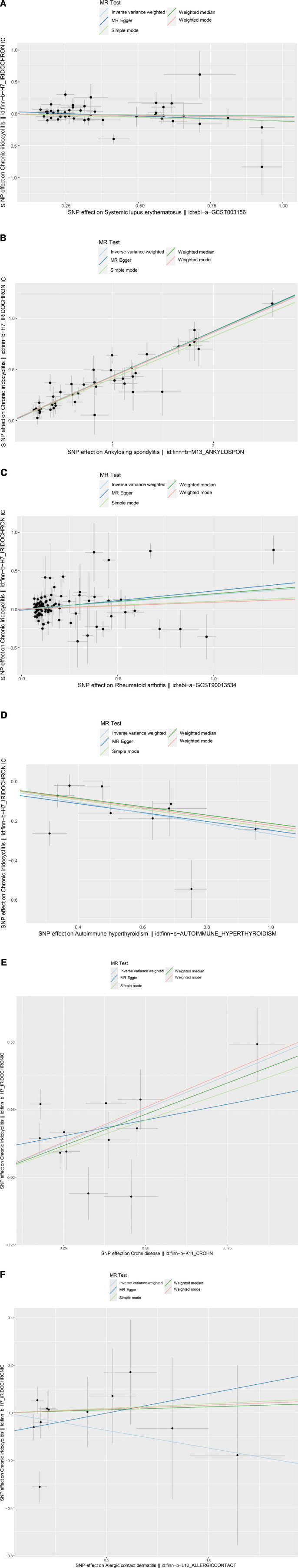
Genetic association of iridocyclitis with immune-related diseases: (A) with systemic lupus erythematosus; (B) with ankylosing spondylitis; (C) with rheumatoid arthritis; (D) with autoimmune hyperthyroidism; (E) with Crohn disease (F) with allergic contact dermatitis. MR = Mendelian randomization, SNP = single nucleotide polymorphisms.

In this study, MR was employed to investigate the causal association between 6 immune-related diseases and iridocyclitis (Fig. 2 and Table 2). During the discovery phase, genetic predictions of AS, RA, GD, psoriasis, and CD were found to be associated with the risk of iridocyclitis. Specifically, genetically predicted AS (odds ratio [OR]: 1.544, 95% confidence interval [CI]: 1.494–1.595, *P* = 1.99 × 10⁻^226^), RA (OR: 1.207, 95% CI: 1.052–1.385, *P* = .003), and CD (OR: 1.654, 95% CI: 1.263–2.166, *P* = 2.54 × 10⁻⁶) were linked to a higher risk of iridocyclitis. Conversely, higher genetically predicted GD (OR: 0.763, 95% CI: 0.674–0.865, *P* = .0002) was associated with a reduced risk of iridocyclitis.

Moreover, findings from the IVW method indicated that SLE and ACD might exert a protective effect on iridocyclitis; however, the results were not statistically significant, and thus a causal relationship between SLE or ACD and iridocyclitis could not be confirmed (Fig. 2).

### 3.3. Sensitivity analyses

Heterogeneity was detected among the SNPs associated with immune-related diseases, although none of the MR-Egger regression intercepts significantly deviated from the null. The horizontal pleiotropy test results indicated that no significant *P* values were observed between SNPs for the aforementioned immune disorders and iridocyclitis in this MR analysis.

**Figure 3.**
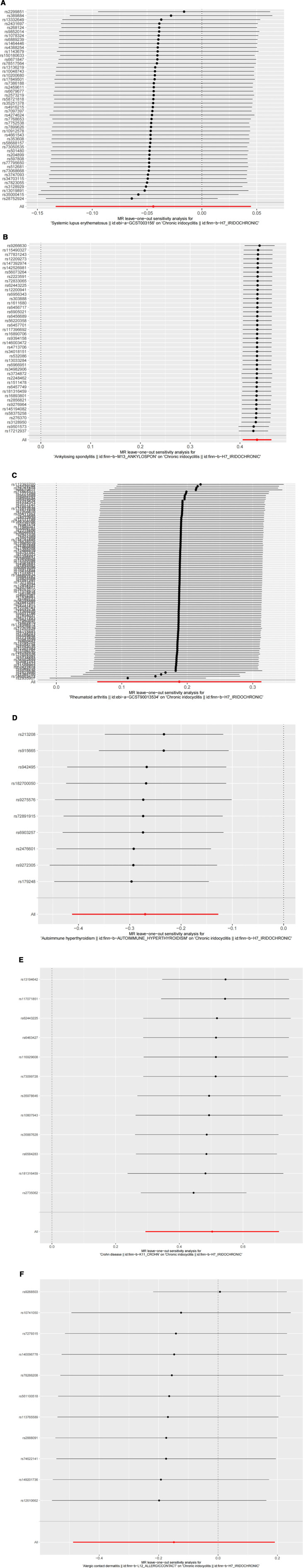
Leave-one-out analysis of the causal effect of immune-related diseases on iridocyclitis: (A) systemic lupus erythematosus; (B) ankylosing spondylitis; (C) rheumatoid arthritis; (D) autoimmune hyperthyroidism; (E) with Crohn disease (F) with allergic contact dermatitis.

Additionally, the leave-one-out analysis revealed no substantial differences in the causal estimations of AID on iridocyclitis, suggesting that none of the identified causal associations were driven by any single IV (Fig. 3).

## 4. Discussion

In this study, we employed MR to explore the causal relationships between 6 immune-related diseases and iridocyclitis. Our findings offer novel insights into the genetic foundations of iridocyclitis, a condition characterized by inflammation of the uvea and iris, often linked to systemic inflammatory disorders.

We identified 230 single SNPs significantly correlated with various immune-related diseases, which served as IVs in our analysis. The use of robust genetic instruments enhances the reliability of our causal estimates. Notably, our results reveal that genetically predicted AS, RA, and CD are associated with an elevated risk of iridocyclitis. AS demonstrated the strongest association, emphasizing potential shared inflammatory pathways between AS and iridocyclitis. The significant association between genetically predicted RA and iridocyclitis suggests considerable overlap in genetic susceptibility. Similarly, the positive association of CD with iridocyclitis underscores the role of gut inflammation in uveitis, aligning with previous clinical observations of this connection. Conversely, GD was linked to a reduced risk of iridocyclitis, indicating a potential protective effect or the involvement of distinct inflammatory mechanisms. This inverse relationship might suggest that the immune response in GD could counteract the pathways leading to iridocyclitis, warranting further mechanistic studies to elucidate this phenomenon.

The association between iridocyclitis and AIDs is deeply intertwined with genetic factors, highlighting shared immunopathogenic mechanisms.^[16]^ Iridocyclitis often coexists with autoimmune conditions such as RA, AS, and CD, pointing to common genetic predispositions, particularly involving HLA alleles and dysregulated immune responses.^[17]^ Genetic variants related to cytokine signaling, such as those in IL-17, IL-23R, and TNF-α pathways, play a significant role in both systemic autoimmune disorders and the local inflammation observed in iridocyclitis.^[18,19]^ These shared genetic and immunological pathways suggest a complex interplay between systemic immune dysregulation and ocular inflammation, emphasizing the need for further research to unravel the genetic underpinnings of iridocyclitis in the context of AID.

Both AS and iridocyclitis share a strong genetic association with the HLA-B27 gene. Approximately 90% of patients with AS are HLA-B27 positive, and up to 50% of patients with HLA-B27-associated anterior uveitis (including iridocyclitis) also have AS. This shared genetic predisposition points to a common pathophysiological pathway involving the HLA-B27 antigen.^[20,21]^ Several SNPs are associated with both AS and iridocyclitis, particularly in genes related to immune system regulation and inflammatory responses. For instance, SNPs in genes such as IL-23R and ERAP1 have been implicated in both conditions. The Th17 subset of T-helper cells, which secrete IL-17, plays a crucial role in the pathogenesis of both AS and iridocyclitis.^[22,23]^ The IL-17 pathway is a key driver of chronic inflammation in both diseases. Additionally, the innate immune system,^[24]^ including the activation of macrophages and dendritic cells, is implicated in both AS and iridocyclitis, where these cells release pro-inflammatory mediators and present antigens that exacerbate the inflammatory process.^[25]^

In RA, the HLA-DRB1 gene is significantly linked to disease susceptibility, particularly through alleles containing the “shared epitope” sequence.^[26,27]^ Although iridocyclitis is not as strongly associated with HLA-DRB1 as RA, evidence suggests that other immune-related genes involved in cytokine signaling pathways may overlap between the 2 conditions. These include polymorphisms in genes like IL-2, IL-6, and TNF-α, which are critical in immune responses.^[28]^ The shared genetic predisposition suggests a common autoimmune mechanism, where susceptibility in immune regulation may lead to inflammation in both joints and ocular tissues.^[29]^ At the molecular level, chronic inflammation mediated by pro-inflammatory cytokines characterizes both iridocyclitis and RA. In RA, cytokines such as TNF-α, IL-1β, and IL-6 are pivotal in driving inflammation and joint destruction. Similarly, in iridocyclitis, elevated levels of TNF-α and IL-6 in the aqueous humor suggest a role in ocular inflammation. The Th17 cell pathway,^[30]^ involving IL-17, is a shared inflammatory pathway in both conditions, contributing to persistent inflammation and tissue damage. This connection indicates that the inflammatory processes in RA could potentially exacerbate or trigger ocular inflammation, leading to iridocyclitis.

Iridocyclitis, and CD share genetic predispositions that underline their autoimmune nature. Both conditions are linked to specific genetic loci that influence immune regulation. In CD, the NOD2/CARD15 gene is a well-established risk factor, playing a crucial role in innate immune responses to bacterial antigens.^[31]^ Similarly, polymorphisms in genes like IL-23R and TNFSF15 have been associated with both CD and uveitis, suggesting a common genetic framework that predisposes individuals to inflammatory responses.^[32]^ These shared genetic factors highlight a potential link between gastrointestinal and ocular inflammation, driven by immune system dysregulation. CD is marked by the overproduction of pro-inflammatory cytokines such as TNF-α, IL-12, IL-17, and IL-23, which are crucial in maintaining intestinal inflammation.^[33,34]^ Similarly, in iridocyclitis, elevated levels of TNF-α and IL-6 contribute to uveal inflammation. The Th17 cell pathway, known for producing IL-17, serves as a common mechanism in both conditions, promoting chronic inflammation and tissue damage. This suggests that similar immune dysregulation might lead to concurrent inflammation in the gut and the eye, linking the 2 diseases through shared molecular pathways.

While direct evidence of a protective role of GD against iridocyclitis is limited, several studies and hypotheses offer potential insights into molecular mechanisms and genetic associations that could contribute to such an observation.^[35,36]^ It is possible that the immune dysregulation in GD, which primarily targets the thyroid, might create a systemic immune environment that lowers the likelihood or severity of uveitis, including iridocyclitis. This could be due to alterations in cytokine profiles or increased activity of regulatory T cells (Tregs), which might inadvertently protect against certain inflammatory ocular responses.^[37]^ Additionally, the presence of autoantibodies in GD, which primarily target thyroid tissues, could theoretically modulate other immune system components, potentially affecting susceptibility to other autoimmune conditions like iridocyclitis.^[38]^ It is also hypothesized that the immune system’s focus on the thyroid gland in GD might divert autoimmune activity, reducing the likelihood of concurrent attacks on ocular tissues.

Although the IVW method indicated a potential protective effect of SLE and ACD on iridocyclitis, these associations did not reach statistical significance. The absence of a causal relationship suggests that these conditions may not share substantial genetic pathways with uveitis, or it may reflect a lack of sufficient power in our study to detect such effects. We observed no significant heterogeneity among SNPs associated with immune-related diseases; the leave-one-out analysis confirmed that the causal associations were not influenced by any single SNP, thereby reinforcing the robustness of our findings.

This study effectively applies MR to explore causal links between 6 immune-related diseases and the risk of iridocyclitis, with significant associations identified for AS, RA, GD, and VD. The use of 230 SNPs, carefully selected based on stringent MR assumptions, strengthens the causal inferences drawn. The application of the IVW method further enhances the precision of the results. However, the study’s conclusions are tempered by several limitations, including reliance on existing GWAS data, and limited generalizability to diverse populations. The absence of individual-level data precluded the possibility of conducting stratified analyses by sex, age, or other demographic factors. Additionally, since the findings are derived from a European population, the causal relationships identified may be influenced by racial bias, necessitating confirmation in genetically diverse populations. Moreover, the MR approach has inherent limitations in the inferred relationships between exposure factors and outcomes.^[39]^ These include the potential failure to establish robust genotype-phenotype or genotype-disease associations, confounding in genotype-phenotype-disease links, issues of pleiotropy and gene multifunctionality, as well as challenges related to canalization, developmental compensation, and the absence of suitable polymorphisms for studying specific modifiable exposures. Future studies should aim to validate these findings across different populations, explore gene-environment interactions, and conduct longitudinal research to capture the dynamic relationship between immune-related diseases and iridocyclitis over time. Longitudinal and functional studies are crucial to validating our findings and elucidating the causal pathways involved. Expanding genetic datasets and incorporating more diverse populations could also enhance the generalizability of our results.

## Acknowledgments

Thank Jinming Zhao for the help and support in statistical analysis.

## Author contributions

**Data curation:** Yao Yao.

**Methodology:** Yao Yao.

**Visualization:** Yao Yao.

**Writing—original draft:** Yao Yao.

**Supervision:** Qian Wang, Wenbin Wei.

**Writing—review & editing:** Qian Wang, Wenbin Wei.

**Project administration:** Wenbin Wei.
